# Elevated Lipoprotein(a) Levels and Massive Ruptured Root and Ascending Aortic Aneurysm

**DOI:** 10.1016/j.jaccas.2025.104921

**Published:** 2025-09-03

**Authors:** Shubh K. Patel, Syed M. Ali Hassan, Nitish K. Dhingra, Raj Verma, Subodh Verma

**Affiliations:** a Temerty Faculty of Medicine, University of Toronto, Toronto, Ontario, Canada; b Division of Cardiac Surgery, St Michael's Hospital of Unity Health Toronto, Li Ka Shing Knowledge Institute, Toronto, Ontario, Canada; c Department of Surgery, University of Toronto, Toronto, Ontario, Canada; d School of Medicine, Royal College of Surgeons in Ireland, Dublin, Ireland; e Department of Pharmacology and Toxicology, University of Toronto, Toronto, Ontario, Canada

**Keywords:** ascending aortic aneurysm, lipoprotein(a), mechanical Bentall procedure

## Abstract

**Background:**

Rupture of a root and ascending aortic aneurysm is a rare, life-threatening condition requiring prompt recognition and surgical intervention. Elevated lipoprotein(a) levels have been implicated in vascular pathology but are less studied in thoracic aneurysms.

**Case Summary:**

A 61-year-old man who presented with severe dyspnea and chest tightness was found to have a 7.3-cm ruptured root and ascending aortic aneurysm with elevated lipoprotein(a) (233 nmol/L). He underwent emergency surgery, including a mechanical Bentall procedure, ascending aortic and hemiarch replacement, and bilateral pericardiectomy. At follow-up, he was clinically stable with a well-seated valve conduit and no complications.

**Discussion:**

This case suggests a potential association between elevated lipoprotein(a) and thoracic aortic aneurysm rupture, possibly linked its proatherogenic and proinflammatory properties in vascular pathology. Further investigations are needed to clarify its role in aneurysm risk stratification.

**Take-Home Messages:**

Elevated lipoprotein(a) may contribute to thoracic aortic aneurysm progression. Its role in risk stratification warrants further investigation.

## Graphical Abstract

**Figure:**
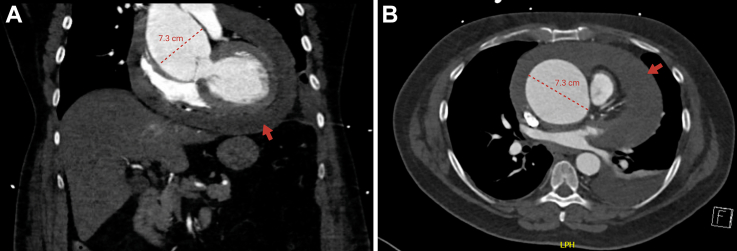

## History of Presentation

**Figure 1:**
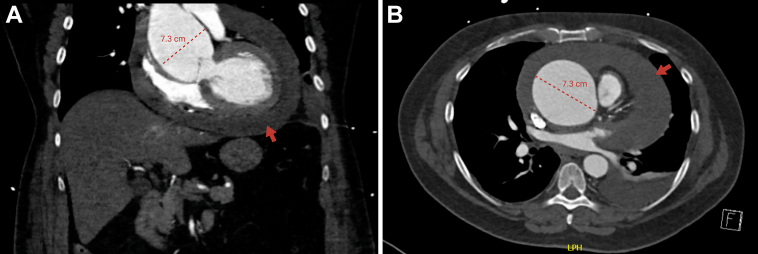
Preoperative Contrast-Enhanced Computed Tomography (A) Coronal and (B) axial views demonstrating a large ascending aortic aneurysm (7.3 cm) with an associated pericardial effusion (red arrow).

A 61-year-old man presented to the emergency department at a community hospital with complaints of severe shortness of breath and a sensation of chest pressure. Two weeks prior, he had experienced severe chest pain, which persisted as a feeling of ongoing chest tightness. On initial evaluation, he appeared stable but reported progressive worsening of symptoms. A computed tomography (CT) scan demonstrated a large root and ascending aortic aneurysm measuring 7.3 cm in diameter, and a large pericardial effusion (Figures 1). Emergent pericardiocentesis drained bloody fluid, relieving tamponade, and confirmed a rupture of the root and ascending aortic aneurysm. Recognizing the life-threatening nature of the aneurysmal rupture, he was emergently transferred to our site for emergent surgical intervention. Consent was obtained from the patient for publication of this case report.

**Take-Home Messages**

• Elevated lipoprotein(a) [Lp(a)] may contribute to the progression and rupture of thoracic aortic aneurysms.
• Further research is needed to determine whether Lp(a) has a causal role and prognostic value in thoracic aortic aneurysm management.

## Past Medical History

The patient had chronic obstructive pulmonary disease, hypertension, dyslipidemia, prediabetes (glycosylated hemoglobin: 6.2%), and known coronary artery disease with a 70% proximal right coronary artery (RCA) stenosis. On admission, his heart rate was 79 beats/min, blood pressure was 143/98 mm Hg (right arm), and weight was 86.3 kg. He was an active smoker with no known allergies or significant family history. His medications included tiotropium bromide, telmisartan, rosuvastatin, salbutamol, prednisone, and ticagrelor.

## Differential Diagnosis

The initial differential diagnosis included aortic dissection, acute coronary syndrome, pericardial tamponade, pulmonary embolism, and ruptured ascending aortic aneurysm. Acute coronary syndrome was deemed unlikely. Pericardial tamponade because of rupture of the root and ascending aortic aneurysm was confirmed by echocardiography and pericardiocentesis, which revealed bloody effusion. Pulmonary embolism was excluded via CT pulmonary angiography. Imaging confirmed a massive root and ascending aortic aneurysm with evidence of rupture and tamponade.

## Investigations

**Figure 2:**
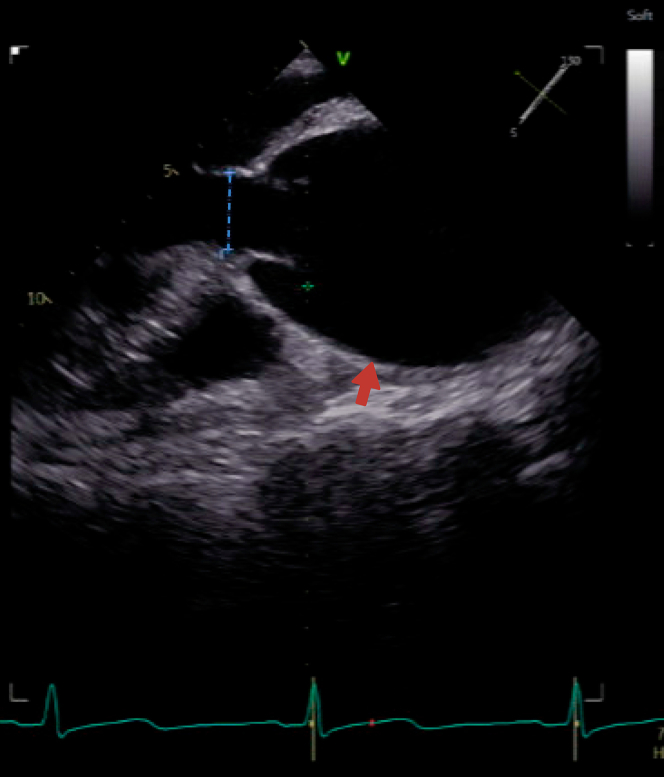
Preoperative Echocardiogram Parasternal long-axis view demonstrating a dilated aortic root and ascending aortic aneurysm (red arrow). The aortic annulus is indicated (blue dotted line).

Initial imaging included transthoracic echocardiography, showing a large pericardial effusion measuring 3.4 cm, significant hemodynamic compromise, and a markedly dilated ascending aorta measuring 7.1 cm, suggestive of aortic rupture (Figure 2). Moderate aortic regurgitation was also noted. A CT angiogram confirmed a massive root and ascending aortic aneurysm measuring 7.3 cm, likely because of aortic rupture with a large pericardial effusion and no dissection flap. Preoperative laboratory tests revealed an elevated serum lipoprotein(a) [Lp(a)] level of 233 nmol/L, along with hemoglobin of 122 g/L, platelets of 397 × 10^9^/L, and creatinine of 63 μmol/L. Liver function tests were normal, and all other laboratory results were unremarkable.

## Management

The patient underwent emergency surgery for a ruptured root and ascending aortic aneurysm measuring 7.3 cm with tamponade. A mechanical Bentall operation was performed using a 23-mm valved conduit, reimplantation of the left and right coronary arteries, and replacement of the ascending aorta and hemiarch with a 26-mm Dacron graft. Deep hypothermic circulatory arrest at 25 °C was used for 19 minutes, with antegrade cerebral perfusion delivered via the right axillary artery for 16.5 minutes.

A saphenous vein graft was harvested for an attempted revascularization of the 70% lesion in the RCA; however, the RCA was encased in inflammatory and fibrous tissue because of the hematoma, preventing adequate exposure. Consequently, revascularization was not performed, and a plan was made for medical management and staged percutaneous coronary intervention.

Postoperatively, the patient experienced severe coagulopathy, likely exacerbated by preoperative ticagrelor use, necessitating the administration of multiple blood products, including red blood cells (Canadian Blood Services), platelets (Canadian Blood Services), fresh frozen plasma (Canadian Blood Services), fibrinogen (Fibryga®, Octapharma Canada Inc.), and factor VII concentrate (NovoSeven® RT, Novo Nordisk A/S). Persistent bleeding required the chest to remain open, packed with Raytec sponges (Accu-Sorb® X-ray–detectable gauze sponges; Medline Industries), for delayed closure the following day. On postoperative day (POD) 1, the patient returned to the operating room for chest closure. All packing materials were removed, and meticulous hemostasis was achieved. The mediastinum was irrigated with saline and bacitracin, and the sternum was closed with stainless steel wires. The patient was stabilized with inotropic support and transferred to the cardiovascular intensive care unit.

Postoperative transthoracic echocardiogram on POD 5 revealed a well-seated mechanical prosthetic valve in the aortic position, with trace intravalvular regurgitation. Left ventricular systolic function was preserved with an ejection fraction of 58%, whereas the right ventricle demonstrated borderline reduced systolic function. The mean gradient across the valve was 19 mm Hg. On POD 7, a CT confirmed stable postoperative changes, with persistent but reduced pericardial and pleural effusions, and a well-positioned ascending aortic graft without any complications. The patient mobilized well under physical therapy, was weaned off supplemental oxygen, and was transitioned to oral warfarin therapy. He was discharged in stable condition with a plan including warfarin therapy, short-term aspirin, endocarditis prophylaxis measures, smoking cessation interventions, and ongoing management of his cardiovascular risk factors.

## Discussion

We present the case of a 7.3-cm ruptured root and ascending aortic aneurysm in a patient with elevated levels of Lp(a), highlighting the potential role of Lp(a) in the development and progression of ascending aortic aneurysms. Although this patient has known cardiac risk factors of hypertension, smoking, and dyslipidemia, the elevated Lp(a) level is an additional potential risk factor that may be associated with ascending aortic aneurysm development and rupture.

Lp(a) is a cholesterol-rich, proatherogenic, and proinflammatory lipoprotein consisting of apolipoprotein B100 linked to apolipoprotein(a).^1^^,^^2^ Plasma Lp(a) levels vary significantly among people, with approximately 20% having high levels (>50 mg/dL), mainly because of genetic factors in the *LPA* gene.^1^ Elevated Lp(a) has been implicated in various vascular pathologies,^1^^,^^3^ promoting chronic vascular inflammation through endothelial activation and extracellular matrix degradation.^4^

Lp(a) localizes predominantly to the intima and subintima, where it adheres to extracellular matrix components such as proteoglycans (eg, decorin) via lysine-binding sites in apolipoprotein(a).^4^ Unlike low-density lipoprotein, Lp(a) binds more avidly to arterial walls and accumulates early in atherogenesis.^4^ Once deposited, it triggers recruitment of monocytes and promotes expression of adhesion molecules like vascular cell adhesion molecule 1, intercellular adhesion molecule 1, and E-selectin on endothelial cells, thus enhancing leukocyte infiltration. Macrophage internalization of Lp(a), especially when oxidized, leads to foam cell formation and release of inflammatory cytokines such as interleukin-1β, interleukin-6, tumor necrosis factor-α, and chemokines like monocyte chemoattractant protein-1 and interleukin-8. These immune responses activate matrix metalloproteinases, which degrade key structural proteins—elastin and collagen—in the aortic media, leading to cystic medial necrosis, elastin fragmentation, smooth muscle cell loss, and proteoglycan pooling.^3^, ^4^, ^5^ Simultaneously, Lp(a) promotes oxidative stress and endothelial dysfunction, accelerating cell senescence, disrupting endothelial barrier integrity, and impairing nitric oxide–mediated vasodilation.^4^ In vascular smooth muscle cells, Lp(a) inhibits transforming growth factor-β activation, enhancing proliferation and reducing autocrine inhibitory feedback on medial remodeling. Additionally, Lp(a) and its oxidized phospholipids may increase extracellular vesicle–mediated calcification in vascular smooth muscle cells, potentially contributing to medial stiffness and aneurysm vulnerability. Together, these mechanisms may contribute to aneurysm formation and rupture.

Although extensive data support the role of elevated Lp(a) in abdominal aortic aneurysm and atherosclerosis,^1^^,^^6^ the evidence for its involvement in thoracic aortic aneurysm remains comparatively weaker and less definitive. Clinical studies illustrate this distinction, with Schillinger et al^7^ demonstrating significantly higher levels of Lp(a) in patients with abdominal aortic aneurysms compared with healthy control patients, suggesting a strong link between Lp(a) and abdominal aneurysm. However, their findings indicated that this association was less pronounced in thoracic aortic aneurysms. Chumachenko et al^8^ similarly found that Lp(a) is infrequently present in the walls of ascending aortic aneurysms, indicating that Lp(a) might not play a significant role in these aneurysms compared with other vascular diseases.

However, a cross-sectional echocardiographic study of 513 hospitalized adults found elevated Lp(a) independently associated with greater diameters at the sinuses of Valsalva (β = 0.330, *P* = 0.002) and sinotubular junction (β = 0.253, *P* = 0.023) among hypertensive, but not normotensive, patients.^9^ Lp(a) also predicted sinuses and ascending aorta dilatation (odds ratio: 1.006 for both; *P* = 0.002 and *P* = 0.035, respectively), with a linear risk relationship observed above 72 mg/dL. This supports a potential role for Lp(a) in thoracic aortic aneurysm development, particularly in the context of coexisting hypertension.

The rapid expansion and rupture of the aneurysm in this case may suggest a contributory role for Lp(a) in the underlying pathophysiological processes; however, causality remains unconfirmed. Although biological plausibility is strong and supported by experimental and some clinical data, robust longitudinal or mechanistic human studies specifically linking Lp(a) to thoracic aortic aneurysm rupture are lacking. Current clinical guidelines for the management of aortic aneurysms do not specifically address the role of Lp(a) measurement, reflecting these ongoing knowledge gaps.^10^ Although routine measurement of Lp(a) is not currently part of risk stratification for thoracic aortic disease, these findings raise the question of whether Lp(a) could serve as a useful biomarker in selected patients. Further research is warranted to evaluate whether a causal relationship exists between elevated Lp(a) levels and the development, progression, or rupture of ascending aortic aneurysms.

## Follow-Up

At 6-week follow-up, the patient was doing well and was active without any symptoms. Home blood pressure was well controlled at 120s/70s mm Hg, and he was hemodynamically stable with a heart rate of 72 beats/min in sinus rhythm. Postoperative echocardiogram confirmed a well-seated mechanical valve with no complications.

## Conclusions

This case highlights the successful surgical management of a massive ruptured root and ascending aortic aneurysm. The markedly elevated Lp(a) level may have contributed to the aneurysm's progression and rupture; however, larger studies are needed to clarify whether Lp(a) plays a causal role or offers meaningful prognostic value beyond established cardiovascular risk factors.

## Funding Support and Author Disclosures

Dr Dhingra reports statistical support from Amarin, Boehringer Ingelheim, Eli Lilly, Lexicon Pharmaceuticals, and Sanofi. Dr. Verma holds a Tier 1 Canada Research Chair in Cardiovascular Surgery; and reports receiving grants and/or research support and/or speaking honoraria from Amgen, AstraZeneca, Bayer, Boehringer Ingelheim, Canadian Heart Research Centre, Canadian Medical and Surgical Knowledge Translation Research Group, Eli Lilly, HLS Therapeutics, Humber River Health, Janssen, Merck, Novartis, Novo Nordisk, Pfizer, PhaseBio, S & L Solutions Event Management Inc, Sanofi, and Sun Pharma. All other authors have reported that they have no relationships relevant to the contents of this paper to disclose.
